# Effect of benazepril, robenacoxib and their combination on glomerular filtration rate in cats

**DOI:** 10.1186/s12917-016-0734-4

**Published:** 2016-06-23

**Authors:** Jonathan N. King, Alessandro Panteri, Melanie Graille, Wolfgang Seewald, Gabriele Friton, Cyril Desevaux

**Affiliations:** Companion Animal Development, Elanco Animal Health, Basel, Switzerland; Preclinical, Elanco Centre de Recherche Santé Animale, St-Aubin, Switzerland; Safety, Elanco Animal Health, Basel, Switzerland

**Keywords:** ACE inhibitor, Benazepril, Cat, Glomerular filtration, NSAID, Robenacoxib, Safety

## Abstract

**Background:**

Combined use of angiotensin-converting enzyme inhibitors and nonsteroidal anti-inflammatory drugs may induce acute kidney injury in humans, especially when combined with diuretics. The objective of this investigation was to evaluate the effects of benazepril, robenacoxib and their combination in healthy cats.

In each of two studies (study 1 followed by study 2), 32 healthy cats were randomised to one of four groups (*n* = 4 male and 4 female cats per group) in a parallel-group design. The groups received orally once daily for 7 days either placebo (control group), benazepril, robenacoxib or benazepril plus robenacoxib. In study 2, all groups received in addition 0.5 mg/kg furosemide twice daily by subcutaneous injection for 7 days.

**Results:**

Benazepril, robenacoxib and their combination were well tolerated as evidenced from lack of clinical signs and no negative effects on body weight, feed consumption and clinical chemistry, haematology and urinalysis variables.

The primary endpoint of the study was the glomerular filtration rate (GFR), which was estimated from the plasma clearance of iohexol. In the absence of furosemide, GFR was significantly higher in cats receiving the combination of benazepril plus robenacoxib compared to the other three groups, and was also significantly higher in females receiving only benazepril compared to the control.

Administration of furosemide induced diuresis, reduced GFR and activated the renin-aldosterone-angiotensin system, evidenced from increased plasma renin activity and plasma aldosterone concentrations. Compared to the control group in cats treated with furosemide, GFR was increased by benazepril (females only) but decreased by robenacoxib (males only). Benazepril, robenacoxib and their combination significantly inhibited the increase in plasma aldosterone induced by furosemide.

**Conclusions:**

The combination of benazepril and robenacoxib was well tolerated and either increased or had a neutral effect on GFR in healthy cats without or with concomitant furosemide. The combination of benazepril and robenacoxib reduced plasma aldosterone concentrations increased by furosemide. It is recommended to test the efficacy and safety of the combined use of benazepril and robenacoxib in cats with clinical disease, notably proteinuric chronic kidney disease.

## Background

Angiotensin-converting enzyme (ACE) inhibitors (ACEIs) are used in the management of various diseases in cats, notably chronic kidney disease (CKD) [1]. Nonsteroidal anti-inflammatory drugs (NSAIDs) are used to treat fever, inflammation and pain. Administration of an ACEI and an NSAID to the same animal may be considered, for example, in cats suffering from both CKD and osteoarthritis. In addition, ACEIs and NSAIDs may have additive efficacy in certain cardiovascular diseases in which inflammation is present, notably proteinuric CKD [2]. Additive effects of ACEIs and NSAIDs in reducing proteinuria have been reported in humans [3].

Negative pharmacodynamic interactions between ACEIs and NSAIDs have been described in humans, however [4]. First, NSAIDs reduce the synthesis of vasodilatory prostaglandins and as a consequence may inhibit the systemic anti-hypertensive efficacy of ACEIs [5]. Second, as a result of reduction in vasodilatory prostaglandins, NSAIDs can constrict the renal afferent arteriole leading to reduced glomerular filtration rate (GFR) and in extreme cases acute kidney injury (AKI). The risk of AKI with NSAIDs is increased by dehydration or reduced blood pressure, for example with general anaesthesia or administration of an ACEI [6].

One of the principal mechanisms of action of ACEIs in slowing the progression of CKD in humans is reduction in glomerular capillary hypertension, mediated via selective dilation of the efferent arterioles in the kidney [1]. As a result of reduced glomerular capillary pressure, ACEIs may reduce GFR in patients with CKD. The benefit to risk ratio of ACEIs in human CKD is positive, however, provided that the reduction in GFR, usually evidenced by increased plasma creatinine concentrations, is not excessive [7]. The combination of vasoconstriction of the renal afferent arterioles by an NSAID and dilation of the efferent arterioles by an ACEI can however produce excessive reduction in GFR potentially leading to AKI [3]. The risk of AKI is further increased by concomitant use of diuretics which themselves reduce blood flow to the kidney via intravascular volume depletion. The danger of the combination of ACEIs, diuretics and NSAIDs in humans was described as a “triple whammy” [8, 9].

To our knowledge, no controlled studies have been reported on the combination of ACEIs and NSAIDs in cats. In contrast to humans, a benefit of ACEIs in slowing the progression of CKD has not been demonstrated in cats, although the ACEI benazepril has been shown to increase GFR while reducing glom erular hypertension in an experimental model of renal insufficiency [10] and to reduce proteinuria in clinical cases of CKD [11]. No significant effect of the NSAID robenacoxib on GFR was reported recently in healthy cats administered furosemide [12]. The effect of the combination of benazepril and robenacoxib on GFR in cats is not known.

The objective of this study was to evaluate the effect of benazepril, robenacoxib and their combination in healthy cats. Two studies were conducted, the first without and the second with concomitant administration of the diuretic furosemide. The primary endpoint was the GFR, which was estimated from the plasma clearance of iohexol.

## Methods

### Studies and approvals

Two studies (termed study 1 and study 2) were conducted using similar study designs, except that in addition in study 2 furosemide was administered and plasma renin activity (PRA) and plasma aldosterone concentration, urine variables including volume and specific gravity (USG), and water intake were measured. Both studies followed a randomised and parallel-group design comparing three treatment groups (benazepril, robenacoxib and their combination) to the control group (Table 1).

**Table 1:** Treatment groups

Group	Treatment (mg/kg) once daily orally		Study 1	Study 2
A	None (placebo, control)	Cohort 1	4 f	2 m & 2 f
		Cohort 2	4 m	2 m & 2 f
B	Benazepril (0.5–1.0)	Cohort 1	4 f	2 m & 2 f
		Cohort 2	4 m	2 m & 2 f
C	Robenacoxib (1.0–2.4)	Cohort 1	4 f	2 m & 2 f
		Cohort 2	4 m	2 m & 2 f
D	Benazepril (0.5–1.0) + Robenacoxib (1.0–2.4)	Cohort 1	4 f	2 m & 2 f
		Cohort 2	4 m	2 m & 2 f

*f* female, *m* male

Studies 1 and 2 were performed respectively in compliance with the registered permit numbers 2011_11_FR and 2012_34E_FR approved by the Swiss cantonal animal welfare committee, and after approval of the protocol by the company Global Animal Welfare Unit. The studies were designed to use the fewest number of animals possible while being consistent with the objectives. The current state of scientific knowledge did not provide acceptable alternatives, *in vitro* or otherwise, to the use of live animals to accomplish the purpose of the studies. The cat is a target species for the test items. Study 2 was initiated only after positive tolerability results were obtained from study 1. All cats were returned to the site’s animal facilities upon completion of the study.

This manuscript was prepared in compliance with the ARRIVE Guidelines Checklist for animal *in vivo* experiments [13].

### Study animals

A total of 48 unneutered European short-haired cats (26 males plus 22 non-pregnant and nulliparous females) were used, with 16 males and 16 females included in each of the two studies. All cats had been bred specifically for research purposes and were owned by the laboratory. A total of 6 males and 10 females were used in both studies, with a washout of 4 months. All cats were healthy and had not received an ACEI or NSAID for at least 2 months prior to each study.

### Housing and management

The acclimatisation phase lasted approximately two weeks and started on day -14 (study 1, Table 2) or -15 (study 2, Table 3). During this phase, the cats were acclimatised to their housing and management and were confirmed to be healthy based on a physical examination, feed consumption and results of clinical chemistry, haematology and urinalyses.

**Table 2:** Schedule of study 1

Category	Cohort 1 study days		Cohort 2 study days	
	Pre-treatment	Post-treatment	Pre-treatment	Post-treatment
Administration of test items	0 to 6		1 to 7	
Haematology, coagulation, clinical chemistry	−14	2, 9	−14	3, 10
Body weight	−15, −8, −1	6	−15, −7, −1	7
Feed consumption	−15 to −8, −7 to −1	0 to 6, 7 to 10	−15 to −8, −7 to 0	1 to 7, 8 to 10
Iohexol concentration (0, 2, 3 & 4 h)	−8	6	−7	7

**Table 3:** Schedule of study 2

Category	Cohort 1 study days		Cohort 2 study days	
	Pre-treatment	Post-treatment	Pre-treatment	Post-treatment
Administration of test items and furosemide^a^	0 to 6		7 to 13	
Haematology, coagulation	−15	5	−15	12
Clinical chemistry	−15, −8	5	−15, −8	12
Urinalysis and water consumption	−14, −7^b^	6	−14, −7^b^	13
Body weight	−16, −13, −9, −6, −1	7	−16, −13, −9, −6, −1, 5	14
Feed consumption	−16 to −1	0 to 6	−16 to 6	7 to 14
Iohexol concentration	−8 (0, 2, 3 & 4 h)	6 (0, 2, 3 & 4 h)	−7 (0, 2, 3 & 4 h)	13 (0, 2, 3 & 4 h)
Plasma renin activity and aldosterone concentration	0^c^ 6 (0 h)	6 (2, 6, 12 & 24 h)	7^c^ 13 (0 h)	13 (2, 6, 12 & 24 h)

*h* hour

^a^Furosemide was administered subcutaneously twice daily on days 0 to 5 (cohort 1) or 7 to 12 (cohort 2) and then as a single dose on the morning of day 6 (cohort 1) or 13 (cohort 2)

^b^Urinalysis at day −7 only if no sample was taken at day −14

^c^For plasma renin activity and plasma aldosterone, the day 0/7 value not used in the analysis

The cats were housed in groups in a climate-controlled building with artificial light from 6.00 to 18.00. The animals were placed in individual cages for up to 5 h daily to record feed consumption, and in study 2 the cats were also placed individually into cages two times for 24 h to measure water consumption and urine volume, and to facilitate collection of urine samples.

Each cat was offered 40–70 g of dried pelleted feed (Purina® Pro Plan Adult, Switzerland) once a day at 12.00 (±1 h), approximately 4 h after dosing with the test items. On the days that blood samples were taken, cats were fed approximately 4 h after dosing with the test items and after the 4 h time point for iohexol determination. Drinking water was supplied *ad libitum*.

### Randomisation and blinding

The cats were assigned at random to one of four groups A, B, C or D (4/gender/group, Table 1), while maintaining homogeneous distribution of body weight where possible. The randomisation was made by the statistician using SAS/STAT® procedure *plan* [14]. Due to the experimental workload, the cats in both studies were divided into two cohorts with the first day of dosing staggered by 1 day in study 1 and 7 days in study 2. In study 1, cohort 1 consisted of all females and cohort 2 all males. In study 2, each cohort consisted of 2 males and 2 females per group.

The study was not blinded. This was judged acceptable since the main endpoints, including the primary endpoint, were objective.

### Test items and furosemide

Benazepril was dosed once daily orally at a minimum dose of 0.5 mg/kg (as the hydrochloride salt) with a range of 0.5–1.0 mg/kg (Fortekor® Flavour 2.5 or 5 mg tablets, Elanco Animal Health, Huningue, France). This is the dosage of benazepril registered for the reduction of proteinuria in cats with CKD in the EU [11].

Robenacoxib was dosed once daily orally at a minimum dose of 1.0 mg/kg with a range of 1.0–2.4 mg/kg (Onsior® 6 mg flavoured tablets, Elanco Animal Health, Huningue, France). This is the dosage of robenacoxib registered for the treatment of acute musculoskeletal disorders in cats in the EU [15].

The seven day treatment time for the test items was selected in order to reach steady state for benazeprilat (steady state plasma concentrations within 2–3 days [16]) and robenacoxib (terminal half-life 1–2 h in blood and ~24 h in peripheral sites of inflammation [17, 18]).

In study 2, furosemide was administered at a target dose of 0.5 ± 0.2 mg/kg twice daily (BID) by subcutaneous injection (Dimazon® Solution for injection, Merck Animal Health, Kenilworth, NJ, USA). Injection was chosen as the route of administration in order to minimise variability in absorption. The manufacturer’s recommended dose of furosemide is 5 mg/kg followed by maintenance at 1-2 mg/kg every 6–8 h. However in a pilot study, furosemide doses of 1 or 2 mg/kg BID for 9 days to cats led to unacceptable reduction in feed intake and body weight. In a second pilot study, 0.5 furosemide mg/kg BID for 7 days produced a significant diuretic effect and compared to 1 mg/kg BID was better tolerated and produced numerically greater stimulation of the renin-angiotensin-aldosterone-system (RAAS), as assessed from PRA and aldosterone.

In order to avoid multiple administrations, benazepril and robenacoxib were dosed together in combinations of whole and part tablets in a single size 00 gelatin capsule. The control group received a placebo consisting of a single empty size 00 gelatin capsule. All treatments were administered at 8.00 ± 1 h (in study 2 furosemide was administered at 8.00 ± 1 h and 20.00 ± 2 h). After dosing, 2.5 mL of water was administered via a syringe to facilitate passage of the capsules into the stomach, and the cats were observed for several minutes to ensure that the capsules had been swallowed.

Doses were determined using the body weight measured on day -1, or day 5 for cohort 2 in study 2 (Table 3). Except for unplanned medical treatments, additional medications were prohibited during the studies.

### Endpoints

The primary endpoint of the study was the GFR, which was estimated from the plasma clearance of iohexol (CLioh).

Secondary endpoints included general clinical monitoring (body weight, clinical examination, feed consumption, clinical chemistry, coagulation and haematology). In study 2, additional secondary endpoints were PRA and plasma aldosterone concentration, water intake, urine volume and USG, and fractional excretions of potassium and sodium.

### Clinical monitoring

Cats were checked at least once daily. A detailed clinical examination was made once during the acclimatisation phase, and then again 96 h after the first dose of the test items and the day following the last dosing.

Cats were weighed in the morning before feeding at least three times in the baseline period and again at the end of the study (Tables 2 and 3). Daily feed consumption was determined as the difference between rations offered and remaining uneaten after approximately 4 h.

Blood samples for clinical pathology (standard haematology, coagulation and plasma clinical chemistry variables) were taken from a cephalic or jugular vein, after feed was removed for at least 12 h, once during the acclimatisation phase (for animal selection and as baseline), once during the treatment phase and in study 1 again after the end of treatment (Tables 2 and 3).

### Glomerular filtration rate (GFR)

The GFR was estimated from the CLioh after intravenous (IV) administration of iohexol (Accupaque® 350, GE Healthcare Buchler GmbH & Co., Braunschweig, Germany). This was performed once during the acclimatisation phase prior to administration of the test item(s) (day -8 or -7) and again after the last treatment.

Cats had free access to water but feed was withheld for at least 12 h before IV administration of iohexol. A target dose of 90 mg of iodine/kg iohexol was administered via an IV catheter placed in a cephalic vein followed by flushing with physiological saline solution. Actual mean (range) doses administered were 90.0 (86.4.2–93.8) iodine/kg in study 1 and 88.9 (81.8–94.3) iodine/kg in study 2. Blood was sampled from a jugular or antebrachial vein (i.e. not a vein used for iohexol administration) in 1.2 mL heparin tubes before and 2, 3 and 4 h after administration of iohexol. Samples were stored on ice until centrifuged at approximately 3,000 g for 15 min at 4 °C. Once centrifuged, plasma was collected and stored at -20 °C before analysis.

Due to breakdown of equipment between studies, plasma iohexol concentrations were analysed using two different methods in studies 1 and 2. Both methods were validated in cat plasma in terms of selectivity, linearity, repeatability, reproducibility and stability according to the document “Guidance for Industry: Bioanalytical Method Validation” (U.S. Department of Health and Human Services et al., 2001, http://www.labcompliance.com/info/links/methods/guidelines.aspx).

In study 1, the endo- and exo-iohexol isomers were quantified separately. Analyses were performed on a high performance liquid chromatography system (Hewlett Packard 1100, Palo Alto, CA, USA) coupled with an ultraviolet detector (Hewlett Packard 1050). The method was linear over the calibration ranges 1 to 80 μg/mL and 2 to 320 μg/mL for endo- and exo-iohexol respectively. Intra-day and inter-day precisions were lower than 9 % for both isomers. The accuracy varied from 98 to 102 %. The lower limit of quantification (LLOQ) was 1 μg/mL with precision 8 % and accuracy 99 % for endo-iohexol, and 2 μg/mL with precision 10 % and accuracy 109 % for exo-iohexol.

In study 2, total plasma iohexol concentrations were measured using an ultra-high performance liquid chromatography system (Acquity UPLC, Waters, Milford, MA, USA) coupled with a triple quadrupole mass spectrometer (Xevo, Waters). The method was validated with a calibration curve ranging from 5 to 400 μg/mL. Intra-day and inter-day precisions were respectively lower than 11 and 12 %. The accuracy varied from 87 to 102 %. The LLOQ was 5 μg/mL with a precision of 7 % and an accuracy of 91 %.

The CLioh was calculated using the methods described by Laroute et al., [19]. In brief, it was first checked that the pre-administration concentrations of iohexol in plasma were below the LLOQ. Next, a log-linear regression of total iohexol concentration versus time was calculated using:

$$ \log\ \mathrm{concentration} = \upalpha + \left(\upbeta\ \mathrm{x}\ \mathrm{time}\right) $$

The area under the curve (AUC) is then:

$$ \mathrm{A}\mathrm{U}\mathrm{C}= \exp \left(\upalpha \right)/\left(\hbox{-} \upbeta \right) $$

Clearance (CL) was calculated as:

$$ \mathrm{C}\mathrm{L} = \mathrm{dose}\ /\ \mathrm{A}\mathrm{U}\mathrm{C} $$

The CL was calculated relative to body weight (BWT) and relative to body surface area (BSA), the latter being estimated by:

$$ \left(\mathrm{B}\mathrm{S}\mathrm{A}\ \left[{\mathrm{m}}^2\right]\right) = 0.00101 \times {\left(\mathrm{B}\mathrm{W}\mathrm{T}\ \left[\mathrm{g}\right]\right)}^{0.71} $$

### Urine variables

In study 2, the cats were placed in cages with specialised litter trays for 24 h on days -14 or -7 and 6 or 13. Water consumption and urine volume were measured and urine samples collected.

The total volume of water consumed over 24 h was calculated from the difference in volume of water provided and remaining in bowls which were suspended above the floor. The volume was checked repeatedly during the day.

Urine was collected from litter trays by using Catrine Pearl Cat Litter® (Kruuse, Langeskov, Denmark).

Urine samples were stored at below -70 °C before analysis for creatinine, potassium and sodium concentrations and USG. The fractional excretions of potassium (FE_K_) and sodium (FE_Na_) were calculated according to the following formulae:

$$ \mathrm{K}\ \mathrm{fractional}\ \mathrm{excretion}\ \left(\%\right)=\frac{\mathrm{Urine}\ \mathrm{K}\ \mathrm{x}\ \mathrm{Plasma}\ \mathrm{Creatinine}}{\mathrm{Plasma}\ \mathrm{K}\ \mathrm{x}\ \mathrm{Urine}\ \mathrm{Creatinine}} $$

$$ \mathrm{N}\mathrm{a}\ \mathrm{fractional}\ \mathrm{excretion}\ \left(\%\right)=\frac{\mathrm{Urine}\ \mathrm{N}\mathrm{a}\ \mathrm{x}\ \mathrm{Plasma}\ \mathrm{Creatinine}}{\mathrm{Plasma}\ \mathrm{N}\mathrm{a}\ \mathrm{x}\ \mathrm{Urine}\ \mathrm{Creatinine}} $$

### Plasma renin activity (PRA) and aldosterone concentration

In study 2, pre-treatment and on the last day of dosing, approximately 1.2 mL of blood was collected in the morning before administration of furosemide and test items (pre-dose) and 2, 6, 12 and 24 h later (post-dose). Blood was collected into pre-cooled tubes containing K_3_ EDTA and aprotinin (500 KIU/mL of blood) and kept on ice until centrifugation at approximately 3,000 g for 15 min at 4 °C. The plasma was collected and stored below -70 °C until analysis. Plasma angiotensin-1 concentrations were measured using an enzyme immuno-assay (Penisula Labs, San Carlos, CA, USA). The PRA was calculated by comparing the concentration of angiotensin-1 obtained after incubation of the samples at +4 °C (absence of renin activity) and +37 °C (presence of renin activity). The angiotensin-1 assay was validated, over the concentration range 30 to 700 pg/mL. Quality control samples at 60, 145 and 500 pg/mL were analysed in each run; the accuracy was within the defined acceptable range of 75–125 % for all 48 samples, with a mean (coefficient of variation (CV%)) accuracy of 96.7 (7.7 %).

Aldosterone was determined in plasma using a Symbiosis liquid chromatography-mass spectrometry system (online SPE, Spark Holland, Emmen, Netherlands). Concentrations of aldosterone are low in urine of cats and therefore plasma concentrations are more relevant clinically [20]. The assay was validated over the concentration range 0.05 to 2.0 ng/mL. Quality control samples at 0.12, 0.6 and 1.8 ng/mL were analysed in each run; the accuracy was within the defined acceptable range of 75–125 % for all 27 samples, with a mean (CV%) accuracy 96.6 (5.7 %).

### Statistical analysis

The software SAS®, Version 9.2 or higher, was used for all analyses [14]. Data are presented as mean (± SD) or range. The experimental unit was results from each individual cat. Two tailed *P* values less than 0.05 were defined as significant with no correction for multiple analyses, in order not to inflate the type II error.

Variables that were analysed only once post-treatment, including GFR, were analysed using analysis of covariance (ANCOVA) with the pre-treatment value as covariate and sex, treatment, and “treatment by sex” interaction as effects (Tables 4 and 6). Various data transformations were attempted and the adequacy of each statistical model was assessed by evaluation of the normality of the residuals (Shapiro-Wilk test, SAS® procedure *univariate*). In all cases, no or a log transformation was selected.

**Table 4:** Results of statistical analyses for selected variables in study 1 analysed by ANCOVA

Variable	Transformation	*P* values in ANCOVA				*P* value for normality	*P* < 0.05 in post-hoc tests*
		Treatment	Sex	Treatment by sex	Baseline		
Iohexol clearance per BWT	None	0.0030*	0.0002*	0.0017*	0.0003*	0.1957	A, B & C < D (all & f)
Iohexol clearance per BSA	None	0.0036*	0.0005*	0.0013*	0.0005*	0.6001	A, B & C < D (all & f), A < B (f)
Body weight	None	0.1823	0.3007	0.0303*	< 0.0001*	0.4012	B < A (m), A < C (f), D < C (all & f)

Only results of variables with a significant (*P* < 0.1) treatment or “treatment by sex” effect are shown. For sex, baseline, normality and post-hoc comparisons, *P* < 0.05 was defined as significant

*BWT* body weight, *BSA* body surface area, *f* female, *m* male

*Results were primarily interpreted from the effect with the *P* value shown in bold

Variables that were measured pre-treatment and multiple times post-treatment were analysed using repeated measures analysis of covariance (RMANCOVA) (Table 7). The pre-treatment value was the covariate and effects were sex, treatment, time and “treatment by sex” and “treatment by time” interactions.

In the event that the *P* value was less than 0.1 for treatment or the “treatment by sex” or “treatment by time” interactions, individual groups were compared by linear contrasts with *P* < 0.05 defined as significant.

SAS® procedure *mixed* was used for the (RM)ANCOVA analyses.

## Results

### Study 1

The 32 cats were aged 8 months to 7 years with bodyweight 2.5–6 kg at baseline. Groups were well matched at baseline. The mean (range) doses of the test items in mg/kg administered orally once daily alone and in combination were respectively 0.69 (0.51–0.88) and 0.70 (0.51–0.98) for benazepril hydrochloride and 1.67 (1.16–2.27) and 1.67 (1.23–2.36) for robenacoxib.

### Clinical monitoring

All test items were well tolerated. One isolated event of bloody loose stools on day 7 was observed in one cat in group B (benazepril) but was not considered to be caused by the test item. No other clinical signs were reported.

There was no significant (*P* > 0.1) treatment effect or “treatment by time” or “treatment by sex” interactions for feed intake or for relevant haematology, coagulation or clinical chemistry variables (data not shown).

Certain variables showed significant (*P* < 0.05) changes from baseline. These included the red cell variables (cell count, haemoglobin and haematocrit) which decreased in all four groups. For coagulation variables, fibrinogen decreased in group B (benazepril) and the activated partial thromoplastin time increased in group B (benazepril) and group D (benazepril plus robenacoxib). For clinical chemistry variables, plasma albumin and calcium decreased and chloride increased in all four groups. There were also increases in plasma potassium (C and D) and sodium (A, B and D), and decreases in aspartate amino-transferase (A and B), phosphorus (A, B and C), total protein (B and D) and urea nitrogen (B and C).

There was a significant “treatment by sex” interaction for body weight, and significant differences between groups in the post-hoc comparisons (Table 4). Differences in change from baseline were small however: B -0.06 kg versus A in males; A -0.08 versus C in females; D -0.05 (all) and -0.08 (females) versus C.

### Glomerular filtration rate (GFR)

**Fig. 1:**
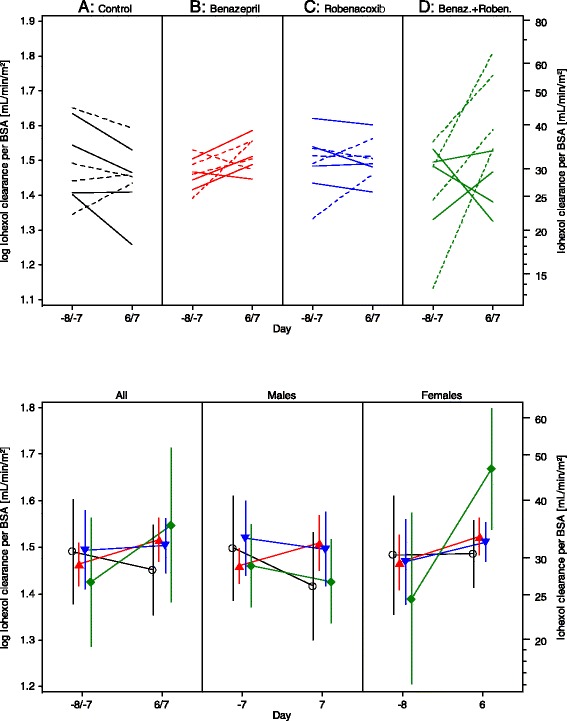
Plasma clearance of iohexol, standardised to baseline body weight (BSA), as an index of glomerular filtration rate in study 1. Upper: Scatter Plots. Data are shown as solid (males) and dotted (female) lines. Lower: Mean (SD) for all cats, and separately for males and females. Control (**A**) –o-; Benazepril (**B**) ; Robenacoxib (**C**) ; Benazepril + Robenacoxib (**D**)

The GFR was assessed from the CLioh. There was low variability in plasma iohexol concentrations (Fig. 1). Similar results were obtained in both studies with CLioh expressed per BWT or BSA. Final conclusions were made using BSA since *P* values for the normality of the data were higher in both studies with CLioh expressed per BSA (Tables 4 and 6).

For CLioh expressed both by BWT or BSA, there were highly significant effects of treatment, sex, “treatment by sex” and baseline in study 1 (Fig. 1, Table 4). In the post-hoc analyses, iohexol clearance was significantly greater in group D (benazepril and robenacoxib) versus the other groups (A, B and C) in all cats and in females. In addition for the BSA endpoint, the clearance was significantly higher in group B (benazepril) compared to the control (A) in females and significance was approached (*P* = 0.051) for males.

The iohexol clearance was highly significantly (*P* < 0.0001) increased compared to baseline in group D (benazepril and robenacoxib) for both BWT and BSA endpoints (all cats and females). The mean change from baseline in CLioh expressed per BSA was: -9.5 %, +12.8 %, +1.3 % and +36.1 % respectively in groups A, B, C and D.

### Study 2

The 32 cats were aged 1–8 years with bodyweight 2.8–7.6 kg at baseline. Groups were well matched at baseline. The mean (range) doses of the test items in mg/kg administered once orally daily alone and in combination were respectively 0.72 (0.51–0.88) and 0.79 (0.58–0.98) for benazepril hydrochloride, and 1.50 (1.02–2.14) and 1.49 (1.05–2.22) for robenacoxib. The mean (range) dose of furosemide was 0.55 (0.50–0.65) mg/kg BID by subcutaneous injection. The furosemide had the desired diuretic effect, producing a marked increase in urine volume (mean +113 %) associated with dehydration, reduced GFR and increase in plasma creatinine concentrations, and activation of the RAAS evidenced from increased PRA and aldosterone concentrations.

*P* values for the primary endpoint and selected (statistically significant) secondary variables are shown for change from baseline (Table 5), ANCOVA (Table 6) and RMANCOVA (Table 7) analyses.

**Table 5:** *P* values for change from baseline for selected variables in study 2

Variable	Sex/time	*P* values for comparison to baseline			
		Group A: Control	Group B: Benazepril	Group C: Robenacoxib	Group D: Benazepril + Robenacoxib
Iohexol clearance per BWT	M	**0.0068 D**	**0.0003 D**	**< 0.0001 D**	**0.0002 D**
	F	**0.0033 D**	0.9475	**0.0248 D**	**0.0003 D**
	Both	**0.0001 D**	**0.0052 D**	**< 0.0001 D**	**< 0.0001 D**
Iohexol clearance per BSA	M	**0.0365 D**	**0.0006 D**	**< 0.0001 D**	**0.0002 D**
	F	**0.0005 D**	0.4843	**0.0030 D**	**< 0.0001 D**
	Both	**0.0002 D**	**0.0024 D**	**< 0.0001 D**	**< 0.0001 D**
Plasma renin activity	Day 6/13 + 2 h	**< 0.0001 I**	**< 0.0001 I**	**0.0012 D**	**< 0.0001 I**
	Day 6/13 + 6 h	**0.0002 I**	**< 0.0001 I**	**0.0166 D**	**0.0002 I**
	Day 6/13 + 12 h	0.1776	**0.0141 I**	**0.0010 D**	0.6073
	Day 6/13 + 24 h	**0.0284 I**	0.8368	**< 0.0001 D**	0.0522 (D)
Plasma aldosterone	Day 6/13 + 2 h	**0.0211 I**	**0.0012 D**	0.5563	**0.0005 D**
	Day 6/13 + 6 h	0.3873	0.0914 (D)	0.9468	**0.0022 D**
	Day 6/13 + 12 h	0.3893	0.1708	0.2046	**0.0262 D**
	Day 6/13 + 24 h	0.5757	**0.0020 D**	**0.0002 D**	**0.0054 D**
Red blood cell count		**< 0.0001 I**	**0.0093 I**	0.0816 (I)	0.0758 (I)
Haematocrit		**0.0004 I**	**0.0109 I**	0.1306	0.0771 (I)
Haemoglobin		**0.0001 I**	**0.0077 I**	0.2113	**0.0298 I**
Prothrombin time		**0.0008 D**	**0.0014 D**	**0.0238 D**	**< 0.0001 D**
Activated partial thromboplastin time		**0.0026 D**	**0.0077 D**	0.2387	0.0515 (D)
Albumin		**< 0.0001 I**	**0.0002 I**	0.1479	0.0595 (I)
Alkaline phosphatase		**0.0121 D**	**0.0001 D**	**0.0429 D**	**< 0.0001 D**
Aspartate aminotransferase		**0.0009 I**	**0.0232 I**	0.1196	**0.0276 I**
Creatinine		**< 0.0001 I**	**< 0.0001 I**	**< 0.0001 I**	**< 0.0001 I**
Total protein		**0.0004 I**	0.0510 (I)	0.9288	0.7448
Urea nitrogen		**< 0.0001 I**	**< 0.0001 I**	**< 0.0001 I**	**< 0.0001 I**
Calcium		**0.0004 I**	**0.0005 I**	**0.0045 I**	0.2532
Chloride		**< 0.0001 D**	**< 0.0001 D**	**< 0.0001 D**	**< 0.0001 D**
Magnesium		**< 0.0001 I**	**0.0003 I**	**0.0033 I**	**0.0022 I**
Potassium		**0.0010 D**	**< 0.0001 D**	**0.0081 D**	**< 0.0001 D**
Sodium		**< 0.0001 D**	**< 0.0001 D**	**0.0188 D**	**< 0.0001 D**
Water consumption		**0.0002 I**	**< 0.0001 I**	**< 0.0001 I**	**< 0.0001 I**
Urine volume		**0.0025 I**	**< 0.0001 I**	**0.0019 I**	**0.0003 I**
Urine specific gravity		**< 0.0001 D**	**< 0.0001 D**	**< 0.0001 D**	**< 0.0001 D**
Fractional excretion potassium		**0.0029 I**	**< 0.0001 I**	**0.0009 I**	**< 0.0001 I**

Only variables with at least one significant (*P* < 0.05) change from baseline are shown

*BWT* body weight, *BSA* body surface area, *F* female, *M* male, *h* hour

*D* decrease, *I* increase (in bold for *P* < 0.05). Non-bold *P* values and D or I in brackets for *P* < 0.1

**Table 6:** *P* values for selected variables in study 2 analysed by ANCOVA

Variable	Transformation	*P* values in ANCOVA				*P* value for normality	*P* < 0.05 in post-hoc tests*
		Treatment	Sex	Treatment by sex	Baseline		
Iohexol clearance per BWT	None	0.0726	0.0534*	0.0451*	0.4365	0.2246	A < B (f), C < A (m), D < B (f & all)
Iohexol clearance per BSA	None	0.0435*	0.2698	0.0300*	0.5357	0.6285	A < B (f), C < A (m), D < B (f & all)
Body weight	None	0.0865*	0.2900	0.1127	< 0.0001*	0.4772	A < C
Albumin	None	0.0066*	0.3807	0.6866	0.1354	0.3174	C & D < A
Total protein	Log	0.0276*	0.7737	0.4227	0.0055*	0.0246*	C & D < A
Chloride	None	0.0245*	0.9598	0.5484	0.2485	0.8120	A < C
Sodium	None	0.0411*	0.3906	0.9749	0.8905	0.4006	A & D < C
Urine specific gravity	None	0.1205	0.4124	0.0914*	0.0544*	0.5961	B < A (f)

Only results of variables with a significant (*P* < 0.1) treatment or “treatment by sex” effect are shown. For sex, baseline, normality and post-hoc comparisons, *P* < 0.05 was defined as significant

*BWT* body weight, *BSA* body surface area, *f* female, *m* male

*Results were primarily interpreted from the effect with the *P* value shown in bold

**Table 7:** *P* values for selected variables in study 2 analysed by RMANCOVA

Variable	Transformation	*P* values in RMANCOVA				*P* value for normality	*P* < 0.05 in post-hoc tests*
		Treatment	Time	Treatment by time	Baseline		
Plasma renin activity	Log	< 0.0001*	< 0.0001*	0.0019*	0.0048*	0.0322	C < A & D (2, 6, 12 & 24 h), D < A (24 h)
Plasma aldosterone	Log	0.0275*	0.0004 *	0.0128*	< 0.0001*	0.1342	B < A (2 h), C < A (24 h), D < A (2, 6 & 12 h), D < C (2 & 6 h)

Only results of variables with a significant (*P* < 0.1) treatment or “treatment by time” effect are shown. For time, baseline, normality and post-hoc comparisons, *P* < 0.05 was defined as significant. There were no significant sex or “sex by treatment” interactions for these variables (data not shown)

*BWT* body weight, *BSA* body surface area, *h* hour

*Results were primarily interpreted from the effect with the *P* value shown in bold

### Clinical monitoring

The test items were tolerated well. Body weight was significantly lower in the control group (A) during treatment compared to the robenacoxib group (C) (Table 6). Incidents of diarrhoea in 4 cats and vomiting in one cat were reported in the pre-test phase but not during dosing with the test items. Skin lesions, attributed to the repeated subcutaneous injection of furosemide or clipping of hair for blood sampling, were observed in one cat in group A and two cats in group D.

Clinical signs attributed to the furosemide included dehydration in 12 animals (4 in group A, 3 in B, 1 in C and 4 in D). Reduced appetite was observed in most cats during furosemide administration and this was reported as inappetence on one or more days for 7 cats (4 in group A, 1 in B and 2 in D). One cat (control group A) was withdrawn from the study due to decreased appetite plus dehydration and prostration.

In the control group (A) there were significant increases (% change) from baseline in: red cell count (+18.0 %), haematocrit (+19.7 %) and haemoglobin (+20.4 %); plasma concentrations of albumin (+0.88 %), creatinine (+39.1 %), total protein (+12.0 %) and urea nitrogen (+15.2 %); water consumption (+73.9 %), urine volume (+113.1 %) and FEk (+75.2 %) (Table 5). There were significant decreases in: prothrombin (-1.8 %) and activated partial thromboplastin time (-4.8 %); plasma concentrations of chloride (-14.9 %), potassium (-10.4 %) and sodium (-4.7 %); and in USG (-6.1 %).

For the variables listed above, there was a significant treatment effect for albumin, total protein, chloride and sodium (Table 6). In the post-hoc comparisons, albumin and total protein were significantly lower in groups C and D versus the control (A), while chloride and sodium were significantly higher in group C versus A.

There was a significant “treatment by sex” interaction for USG, and in the post-hoc comparisons female cats had lower USG in group B (benazepril) compared to the control (A).

### Glomerular filtration rate

**Fig. 2:**
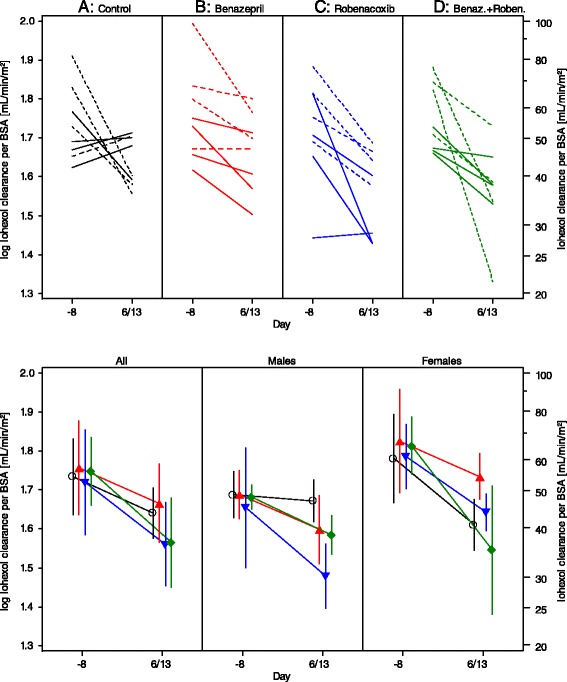
Plasma clearance of iohexol, standardised to baseline body weight (BSA), as an index of glomerular filtration rate in study 2. Upper: Scatter Plots. Data are shown as solid (males) and dotted (female) lines. Lower: Mean (SD) for all cats, and separately for males and females. Control (**A**) –o-; Benazepril (**B**) ; Robenacoxib (**C**) ; Benazepril + Robenacoxib (**D**)

Similar results were obtained with CLioh expressed per BWT or BSA, with final conclusions made using BSA since the *P* value for normality was higher (Table 6). The CLioh decreased significantly from baseline in all groups (Fig. 2, Table 5), attributed to the effect of furosemide. For CLioh expressed per BSA, there were significant treatment and “treatment by sex” effects. In the post-hoc comparisons, CLioh was significantly higher in the benazepril group (B) compared to the control (A, females only) and benazepril plus robenacoxib groups (D, females and all cats). The CLioh was significantly higher in the control group (A) compared to robenacoxib (C, males only).

The mean change from baseline in CLioh expressed per BSA was: -20.4 %, -19.9 %, -31.6 % and -33.7 % respectively in groups A, B, C and D.

### Plasma renin activity (PRA) and aldosterone

**Fig. 3:**
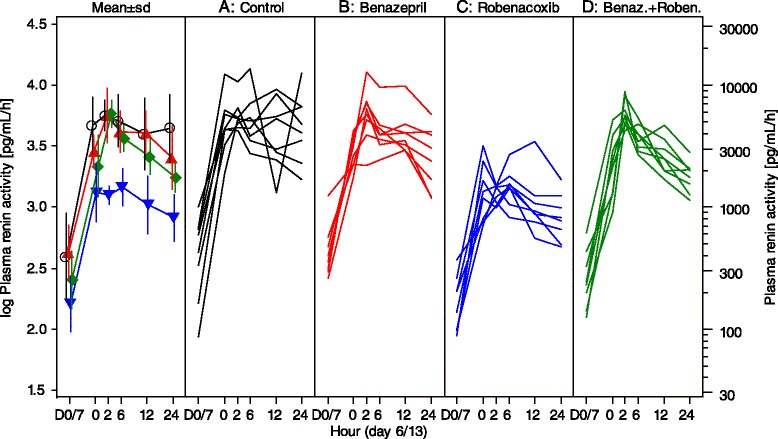
Plasma renin activity (PRA) in study 2. Data are mean (SD) and scatter plots. Test items (benazepril and/or robenacoxib) plus furosemide were administered at time 0 h. D = day. Control (**A**) –o-; Benazepril (**B**) ; Robenacoxib (**C**) ; Benazepril + Robenacoxib (**D**)

**Fig. 4:**
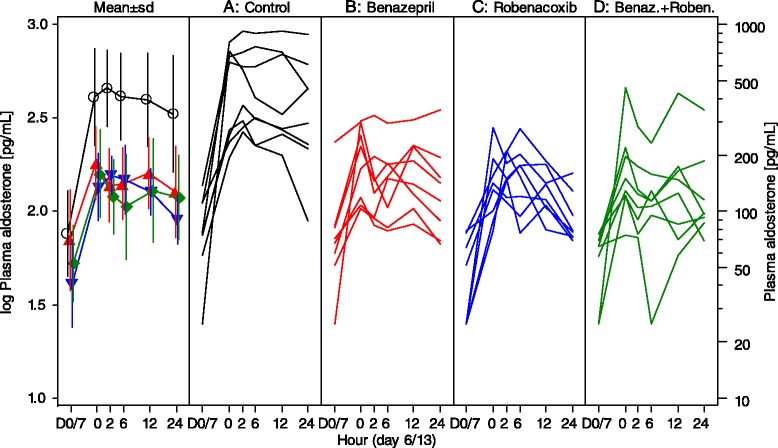
Plasma aldosterone concentration in study 2. Data are mean (SD) and scatter plots. Test items (benazepril and/or robenacoxib) plus furosemide were administered at time 0 h. D = day. Control (**A**) –o-; Benazepril (**B**) ; Robenacoxib (**C**) ; Benazepril + Robenacoxib (**D**)

In the control group (A) there were significant increases from baseline in PRA (at 2, 6 and 24 h after dosing) and plasma aldosterone concentrations (at 2 h) (Figs. 3 and 4, Table 5). At peak effect (2 h), mean values were increased by +1083 % for PRA and by +519 % for aldosterone.

There were significant treatment and “treatment by time” effects for both PRA and aldosterone (Table 7). In the post-hoc analyses for PRA, values were significantly lower in group C versus groups A and D at all time points, and in group D compared to the control group A at 24 h.

In the post-hoc analyses for aldosterone, values were significantly lower at one or more time points in groups B (2 h), C (24 h) and D (2, 6 and 12 h) compared to the control group A. Values were also significantly lower in group D compared to C at 2 and 6 h. Examination of the mean values indicates, however, that all three treatment groups (B, C and D) were associated with approximately equal inhibition of the increase in plasma aldosterone induced by furosemide (Fig. 4).

## Discussion

In humans concomitant administration of ACEIs and NSAIDs can potentially lead to adverse events, notably reduction in GFR and the occurrence of AKI. This risk is worsened by concomitant administration of diuretics, described as a “triple whammy” [8, 9]. In this study in healthy cats, no reduction in GFR was observed with the combination of benazepril and robenacoxib, with and without concomitant administration of the loop diuretic furosemide. Similar findings were reported recently in dogs [21]. The dose of furosemide administered (in study 2) was 0.5 mg/kg BID by subcutaneous injection. This is lower than the dose range of 1–5 mg/kg recommended by the manufacturer, but in pilot studies doses of 1 mg/kg BID and higher produced unacceptable loss of appetite. At the chosen dose, furosemide had the desired diuretic action in study 2, leading in the control group to a significant increase (% change from baseline) in urine volume (+113.1 %) and estimated water consumption (+73.9 %), induced dehydration (haematocrit +19.7 %, total plasma protein +12.0 %), reduced USG (-6.1 %) and renal function (plasma creatinine +39.1 %, GFR -20.4 %), and decreased prothrombin (-1.8 %) and activated partial thromboplastin (-4.8 %) times. The furosemide also led to activation of the RAAS, evidenced by significant increases in PRA and plasma aldosterone concentrations (respectively +1083 % and +519 % at peak effect). In addition, 4 of 12 cats in the control group receiving furosemide had reported inappetence and one cat had to be withdrawn from the study due to decreased appetite plus dehydration. The dose of furosemide was therefore sufficient to induce the necessary pharmacological effects needed for this study to be relevant clinically, and was the highest that could be accepted from an animal welfare perspective. Furosemide reduced GFR significantly and by a mean of 20.4 %. This is a known effect of diuretics, mediated via reduction in perfusion pressure. The diuretic action of furosemide, but with no reduction in GFR, has been demonstrated previously in cats [22].

In this investigation benazepril had no significant effect on GFR compared to the control group in all cats, although GFR was increased in female cats only in both studies 1 and 2. The PRA was increased compared to baseline by a similar extent in the control (A) and benazepril (B) groups in study 2. Increased PRA is a standard effect of ACEIs mediated by reduced synthesis of angiotensin-2 reducing the negative feedback of angiotensin-2 on renin release. The fact that benazepril did not increase PRA, an index of RAAS activation, more than that induced by furosemide alone suggests that the increase in PRA induced by the 0.5 mg/kg BID furosemide dose was already maximal. This conclusion is supported by results from the pilot study in which a dose of 1.0 mg/kg furosemide BID did not increase PRA more than 0.5 mg/kg BID. Finally, benazepril significantly inhibited the increase in plasma aldosterone induced by furosemide. Inhibition of aldosterone synthesis, secondary to reduced synthesis of angiotensin-2, is a known action of ACEIs including in cats [23].

The effects observed with benazepril, with inhibition of the RAAS and reduction of plasma aldosterone concentrations, are consistent with its demonstrated action in reducing proteinuria in cats with CKD [11]. As noted in the introduction, ACEIs as a group have the potential to reduce GFR in (human) patients with heart or kidney disease [7]. In cats with CKD however, no significant increase in plasma creatinine concentrations was observed with benazepril compared to placebo, even at the start of treatment [11], consistent with the finding in this study that benazepril did not reduce GFR. Previously Brown et al., also reported that benazepril increased GFR in cats with nephrectomy-induced chronic renal insufficiency [10].

Administration of robenacoxib without benazepril produced no consistent effects on GFR. The change from baseline in GFR in the robenacoxib versus the control group was respectively +1.3 % versus -9.5 % in study 1, and -31.6 % versus -20.4 % in study 2 (with furosemide). Differences were significantly different for males in study 2. No significant effects of robenacoxib on GFR were reported previously at a high dose (30 mg/kg) in rats [24] or at a clinically relevant dose (2 mg/kg SID or BID orally) in cats [12].

Limited studies on the effect of other NSAIDs on GFR in cats are available. Meloxicam was reported not to change GFR in healthy euvolaemic cats with reduced renal mass [25] or fed a sodium deficient diet [26]. In addition no evidence of deleterious effects on renal function, assessed from plasma creatinine and urea concentrations and lack of adverse events, was reported from field studies in cats receiving carprofen [27, 28], meloxicam [28–30] or robenacoxib [31, 32]. However, in one retrospective study, 48 cases of acute renal failure were reported in cats and 21 of these cases were attributed to the NSAIDs nimesulide (16), tolfenamic acid (3) and ketoprofen (2) [33].

Robenacoxib administered alone significantly inhibited the increase in both PRA and plasma aldosterone concentrations induced by furosemide. Similar results were reported previously with robenacoxib in dogs [21] and other NSAIDs in rats and humans [34, 35]. However no effect of robenacoxib on PRA or aldosterone compared to placebo was reported recently in healthy cats administered furosemide [12].

Many of the renal effects of furosemide are mediated by prostaglandins, and it has been shown in rats and humans that certain NSAIDs inhibit the diuretic, natriuretic and chloruretic effects of furosemide via inhibition of the reabsorption of sodium and chloride in the loop of Henle [35]. There is only weak evidence from this study that robenacoxib inhibited the diuretic action of furosemide; the significant increases from baseline in plasma albumin and total protein concentrations induced by furosemide were significantly attenuated by robenacoxib and the combination of benazepril and robenacoxib, consistent with less dehydration. In addition, robenacoxib alone inhibited the reduction in body weight (which correlated with reduced feed consumption) and plasma sodium and chloride concentrations induced by furosemide. However there were no significant or numerically visible differences between robenacoxib and placebo treated cats for other relevant variables including water consumption, urine volume and USG.

The principal objective of this study was to investigate the tolerability of the combination of benazepril and robenacoxib, notably with concomitant administration of furosemide. The triple administration of an ACEI, diuretic and an NSAID increases markedly the risk of AKI in humans [8, 9]. In this investigation, the combination of benazepril and robenacoxib was tolerated well and had no significant negative effects on GFR compared to the control group. In study 1, in fact, the combination of benazepril and robenacoxib was associated with a significant increase in GFR (+36.1 %) from baseline, compared to a 9.5 % decrease in the control. In study 2, with furosemide, the GFR was significantly lower in the benazepril and robenacoxib combination group compared to the benazepril group, but was not significantly different from the control. Therefore the results do not preclude the possibility that GFR might decline if robenacoxib was administered to a suspectible (e.g. dehydrated) cat which was previously stable with benazepril treatment.

The combination of benazepril and robenacoxib significantly inhibited plasma aldosterone concentrations compared to the control group, with mean values numerically similar to the benazepril alone group but with statistical significance at more time points (2, 6 and 12 versus 2 h). These results support the rationale for investigation of co-administration of benazepril and robenacoxib in certain diseases in cats e.g. proteinuric CKD. Aldosterone is a key mediator contributing to the progression of CHF and CKD in humans [36, 37]. Although higher aldosterone concentrations were detected in cats with hypertension [38], we are not aware of data reliably demonstrating a link between elevated aldosterone concentrations and a poor prognosis in cats. Additive effects of ACEIs and NSAIDs in reducing proteinuria have been described in humans [2, 3].

The principal limitations of the study are listed below.

First, healthy and relatively young cats were studied and the results cannot therefore be simply generalised to cats with diseases such as CKD and osteoarthritis. For example, the residence time of robenacoxib in tissues is markedly prolonged by the presence of inflammation [18] and therefore its duration of action may also be longer in the feline kidney if inflammation is present as compared to the healthy animals used in this study. In addition, the effect of the test items, including on GFR, might be different in the presence of diseases.

Second, the main endpoints in this study were orientated to safety, including GFR to assess the risk of AKI. We did not measure blood pressure, and therefore did not test for a possible interaction of robenacoxib with the anti-hypertensive efficacy of benazepril.

Third, GFR was assessed 2–4 h after dosing with the test items i.e. close to the peak concentrations of plasma benazeprilat (≤2 h) and blood robenacoxib (0.5 h) [16, 17]. The observed effects of benazepril and robenacoxib on GFR therefore presumably represent maximal or close to maximal actions, and different (probably lesser) effects might occur at other times (4–24 h) of the dosing interval.

Fourth, the cats were fed a standard diet and were dosed with the test items in the morning at approximately 8.00. Chronological differences and marked effects of feeding on the RAAS have been reported in dogs [39], and therefore different results might be obtained with other diets or different timings of feeding or dosing.

Fifth, sex and “sex by treatment” interactions were significant for several variables in both studies. The frequency of these interactions was greater in study 2, therefore the weakness in study design in study 1 (all females were included in cohort 1 and all males in cohort 2) did not appear to have a relevant impact on the results. We are not aware of previous reports of genuine gender differences in the effects of ACEIs or NSAIDs on GFR in other species. No sex differences were reported previously in the pharmacokinetics or pharmacodynamics of benazepril [16, 40, 41] or robenacoxib [17, 42] in cats. Therefore we suspect that the observed differences between genders, in addition to isolated events of statistical significance for some chemistry and haematology variables, may be type I errors associated with the multiple statistical analyses with no correction of the alpha value, rather than being genuine biological effects.

Last, the results of this study cannot be simply extrapolated to other ACEIs and NSAIDs since the pharmacology of individual agents differ. Of specific relevance to this study is fact that the active metabolite of benazepril, benazeprilat, is excreted mainly (~85 %) via the biliary rather than renal (~15 %) route in cats [41], and therefore its clearance is maintained even in the presence of moderate renal insufficiency [40]. Similar conclusions might therefore not be obtained for other ACEIs which are mainly or exclusively renally excreted. In addition robenacoxib has a short residence time in the central compartment [17, 18], and therefore is assumed to have short duration pharmacodynamic actions in the healthy kidney. Similar conclusions might therefore not be obtained with NSAIDs with longer half-lives or which are not cyclo-oxygenase (COX)-2 selective. Robenacoxib is a highly selective inhibitor of COX-2 [42], and there is some evidence in humans that the risk of AKI may be lower with selective COX-2 inhibitors compared to non-selective NSAIDs [43].

## Conclusions

The combined administration of benazepril and robenacoxib was well tolerated, and either increased or had a neutral effect on GFR compared to the control group in healthy cats without or with concomitant furosemide. The combination of benazepril and robenacoxib reduced plasma aldosterone concentrations increased by furosemide and therefore might be beneficial in cats with certain diseases, notably proteinuric CKD. It is recommended to test the efficacy and clinical safety of combined administration of benazepril and robenacoxib in cats in field studies.

## Abbreviations

ACE, angiotensin-converting enzyme; ACEI, angiotensin-converting enzyme inhibitor; AKI, acute kidney injury; ANCOVA, analysis of covariance; AUC: area under curve; BID, twice daily; BSA, body surface area; BWT, body weight; CKD, chronic kidney disease; CL, clearance; CLioh, plasma clearance of iohexol; CV, coefficient of variation; F, female; FE_K_, fractional excretion of potassium; FE_Na_, fractional excretion of sodium; GFR, glomerular filtration rate; IV, intravenous; LLOQ, lower limit of quantification; M, male; NSAID, nonsteroidal anti-inflammatory drug; PRA, plasma renin activity; RAAS, renin-angiotensin-aldosterone system; RMANCOVA, repeated measures analysis of covariance; USG, urine specific gravity.
